# Evidence of Direct Exciton Polaron Formation upon Photoexcitation in Wurtzite ZnO

**DOI:** 10.1021/acs.jpclett.6c02424

**Published:** 2026-09-25

**Authors:** Erica P. Craddock, Kathryn E. Knowles

**Affiliations:** Department of Chemistry, 6927 University of Rochester, Rochester, New York 14627, United States

## Abstract

This paper extends the investigation of direct photoexcitation into polaronic states within transition metal oxide materials to ZnO, which has long been understood to form strongly bound excitons even at room temperature. The extent to which these excitons couple to the lattice to form exciton polarons and the nature of this coupling are less clear. Evidence for direct photoexcitation into polaronic states has been reported recently for several transition metal oxides; however, these investigations have been limited to electron or hole polarons. Here, we apply a combination of resonance Raman and temperature-dependent absorption and emission spectroscopies supported by phonon calculations to investigate exciton–phonon coupling in wurtzite ZnO thin films. We find that the A_1_ (LO) and E_1_ (LO) phonons exhibit strong coupling to excitonic transitions. These data are consistent with the direct excitation of intrinsic exciton polarons via coupling to thermally populated phonons within a pristine ZnO lattice.

## Graphical abstract

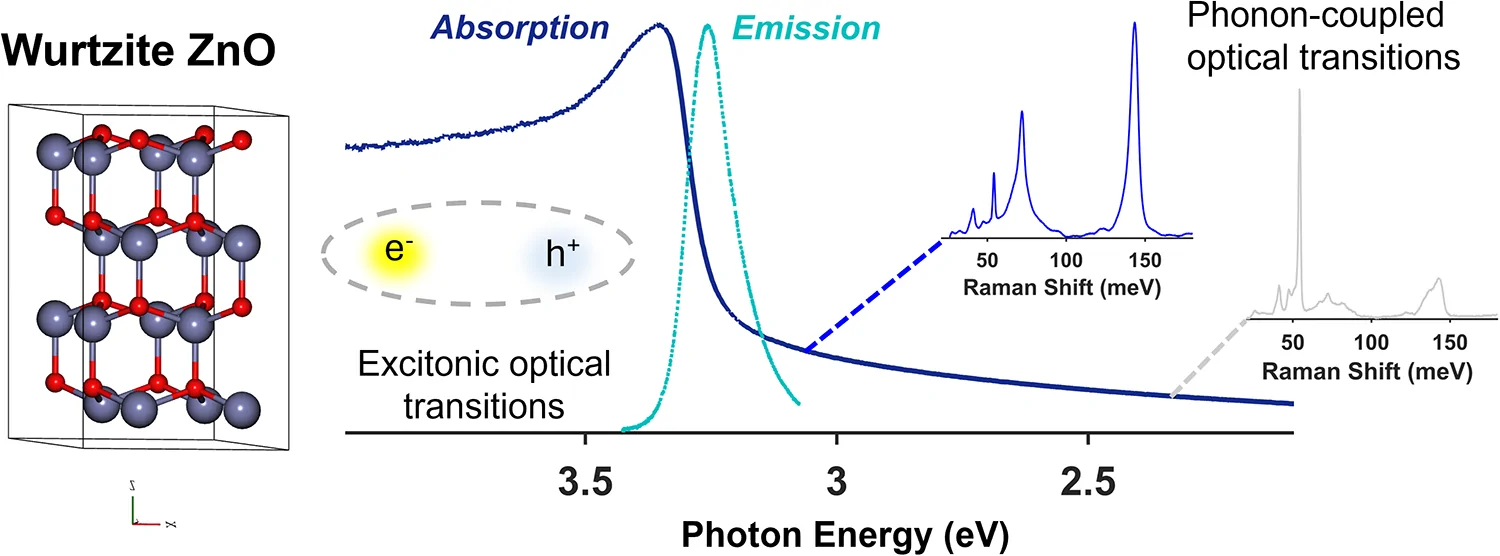

Transition metal oxides are prone to forming self-trapped states arising from the coupling of charge carriers and intrinsic lattice vibrations.^1–4^ Many transition metal oxides are promising for photoapplications, particularly photocatalysis, and the formation of these self-trapped states, termed polarons, upon photoexcitation can greatly impact their performance.^3,4^ Although the coupling of an electron to lattice motion was first described over a century ago,^5–7^ the formation of polarons upon photoexcitation, which simultaneously generates both an electron and a hole, has only recently been recognized as a distinct photophysical process.^4^ Previous work from our group has established a mechanism of direct population of a polaronic state upon photoexcitation via coupling to thermally populated phonon modes in myriad open-shell transition metal oxides.^8–12^ Importantly, resonance Raman analysis reveals that specific intrinsic phonon modes (i.e., unrelated to defects) exhibit enhancement at the onset of optical absorption. The involvement of these modes in band-edge absorption is further corroborated with thermal difference spectroscopy and computational analysis. Here, we extend this framework of thermally populated phonon modes coupling to optical transitions to directly populate polaronic states to ZnO, a transition metal oxide with a full *d*-shell and a well-documented excitonic transition.^13,14^ We treat ZnO as a model system to investigate how excitonic transitions couple to phonon modes to mediate polaron formation.

Decades of research using both spectroscopic and computational approaches to understand the optical properties of ZnO has yielded a vast body of work;^13–28^ however, the exact nature of the lowest-energy excited state in ZnO remains ambiguous. Although the consensus, based on early reports of temperature-dependent absorption and reflection spectra, is that ZnO forms a strongly bound exciton, the extent to which this exciton couples to lattice distortions to form an exciton polaron is less clear.^13–16^ Resonant Raman studies have observed second-order scattering in ZnO,^17,18^ which, when combined with calculated electron–phonon interaction constants, allude to Frölich interactions.^18^ Hsu, et al. use temperature-dependent emission to propose excitonic polarons in ZnO nanorods.^19^ Computational approaches also indicate polaron formation in wurtzite ZnO: Lany and Zunger adapted standard local-density calculations to accurately accommodate polaronic distortions to reveal that cation-site acceptors can bind multiple holes when considering oxygen hole localization in ZnO.^20^ Modeling angle-resolved photoemission spectroscopy (ARPES) measurements of ZnO with varied degrees of hydrogen doping enabled Antonius, et al. to assess electron–phonon coupling.^21^ They find excellent agreement between the simulated undoped ZnO ARPES results and experimental band gap energies in addition to LO phonon branches around 65 meV with strong polar interactions contributing to carrier–phonon coupling. They identify satellite bands associated with the coupled LO phonon branch that appear as an elongated tail, an attribute characteristic of a polaronic state.^21^ However, a recent study by Hua et al. in which ZnO nanoparticles were analyzed with ultrafast UV pump-optical probe spectroscopy and ultrafast electron diffraction found that small hole polarons form on the order of ∼1 ps. These hole polarons then act as defect trap states, resulting in the formation of long-lived and confined exciton polarons with lifetimes > 6 ns.^22^ The trapping of holes at oxygen vacancy sites has also been inferred from transient X-ray spectroscopy of ZnO nanoparticles using above band gap excitation, corroborated by theoretical modeling.^23^ The formation of hole polarons in ZnO is further corroborated with time-resolved electronic sum-frequency generation^24^ and by tracing near-infrared spectral changes after ultraviolet photoexcitation at low temperatures.^28^

Our group has demonstrated that resonance Raman spectroscopy combined with temperature-dependent optical spectroscopy and Hubbard-corrected density functional theory (DFT+*U*) calculations reveals signatures of photoinduced polaron formation in transition metal oxide semiconductors.^4,8–12^ Here, we apply this approach to ZnO. We observe evidence for strong phonon coupling to the excitonic transition of ZnO, which is consistent with previous reports^19,22^ and suggestive of exciton polaron formation. Our data indicate that polaron formation occurs at specific excitation photon energies that coincide with the onset of excitonic absorption and implicate the participation of specific optical phonon modes, namely, A_1_-LO and E_1_-LO, in photogenerated polaron formation. Notably, these data suggest that, as with electron or hole polarons in nonexcitonic transition metal oxides such as α-Fe_2_O_3_, Co_3_O_4_, and ternary spinel ferrites,^8,10,11^ exciton polarons can form directly upon photoexcitation via coupling to thermally populated phonons.^4^ This mechanism could occur in parallel with polaron formation mediated by oxygen vacancy defects implicated in previous work.^20–23,28^

Figure 1 shows imaginary dielectric and emission spectra along with a powder X-ray diffraction pattern of a polycrystalline sample of wurtzite ZnO, all of which were collected at room temperature. The powder X-ray diffraction pattern confrims the sample comprises phase-pure wurtzite ZnO. The dielectric spectrum, which was derived from fitting a series of transmission and reflection spectra to Fresnel equations (see Figure S1 in the Supporting Information), exhibits a sharp feature centered at 3.36 eV, while the emission spectrum comprises a narrow peak centered at 3.26 eV. These features are consistent with previously reported spectra of ZnO and are assigned to excitonic absorption and emission, respectively.

**Figure 1:**
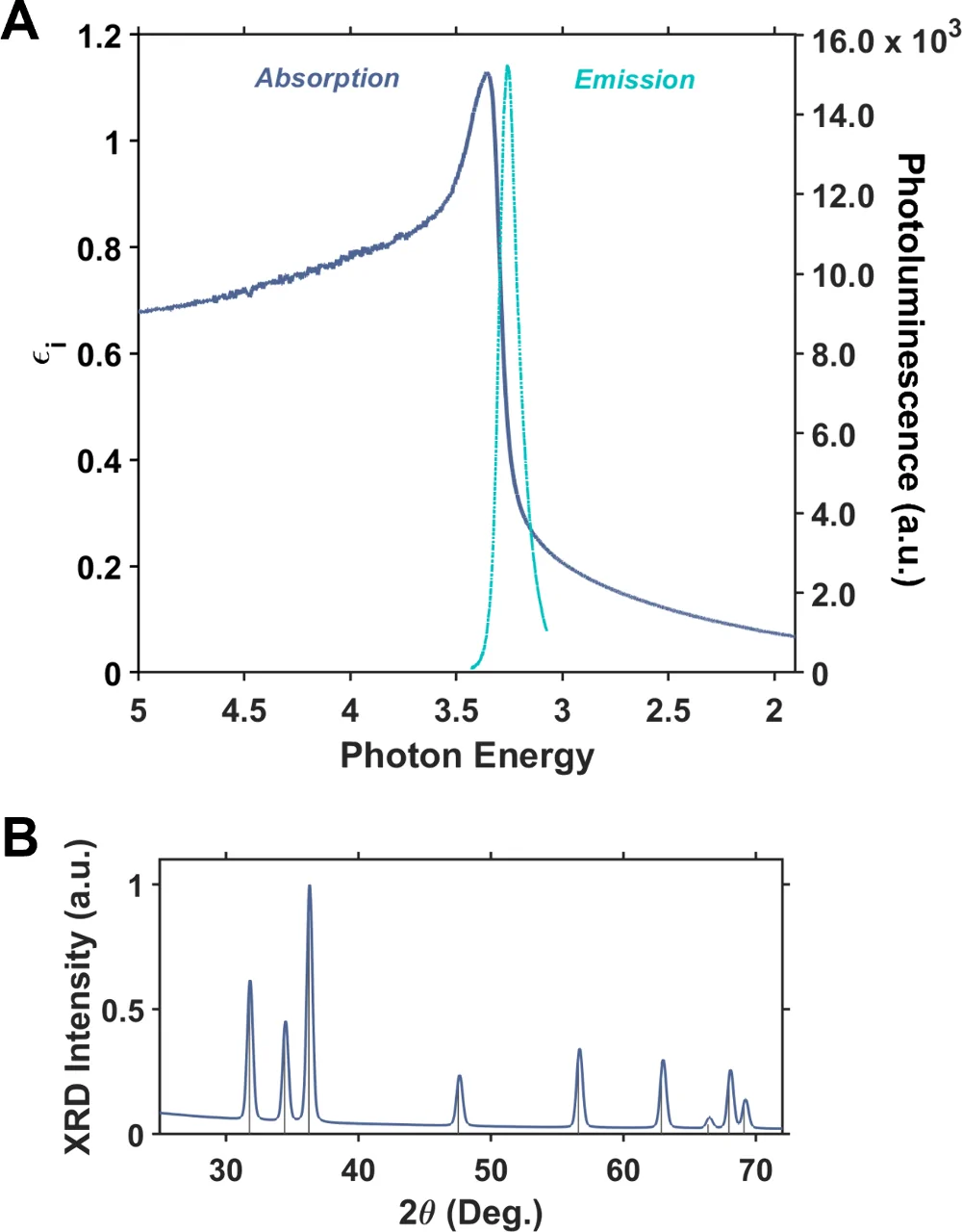
**A)** The imaginary dielectric spectrum of ZnO determined from experimental Fresnel analysis overlaid with an emission spectrum obtained using an excitation energy of 3.49 eV. **B)** Powder X-ray diffraction pattern of a polycrystalline ZnO sample overlaid with standard reflections (ICDD: 04-015-4060).

Interactions between excitons and phonons are known to modulate optical properties;^29^ it is therefore critical to understand the intrinsic optical phonon modes of ZnO and how they couple to its excitonic transition. The phonon bands and corresponding vibrational density of states calculated with Hubbard-corrected density function theory (DFT+*U*) are shown in Figure 2A (see Methods section for details and rationale associated with this computational approach). The displacement vectors associated with each optical phonon mode at the Γ point can be found in the Supporting Information (Figure S2). The blue rectangles in the phonon band structure indicate the energies of fundamental phonon modes of wurtzite ZnO at the Γ point and correspond to the previously reported symmetries of 2E_2_ + E_1_ + A_1_ + 2B_1_.^30^ The hexagonal crystal structure and resulting polarization-dependence lead to both the A_1_ and E_1_ phonons splitting into longitudinal and transverse optical modes, reflected in the vibrational band structure at Γ and the different phonon displacement vectors (Figure S2). The splitting of these phonons is observed experimentally in polarization-dependent Raman spectra of single-crystalline ZnO and previously reported DFT studies.^18,31,32^ ZnO also exhibits a distinct phonon band gap, defined at lower energy by the 34-meV B_1_ mode and at higher energy by the 44-meV A_1_-TO mode. The projected vibrational densities of state demonstrate that when the phonon energy is at or below 34 meV, the displacement vector is dominated by Zn motion. Conversely, when the phonon energy is greater than or equal to 44 meV, the displacement vector is dominated by oxygen motion. With a close look at the vector displacement images of optical phonon modes at Γ, both the A_1_-LO and E_1_-LO modes have minor Zn contributions; however, the overall trend of majority oxygen motion at higher energy modes holds (Figure S2).

**Figure 2:**
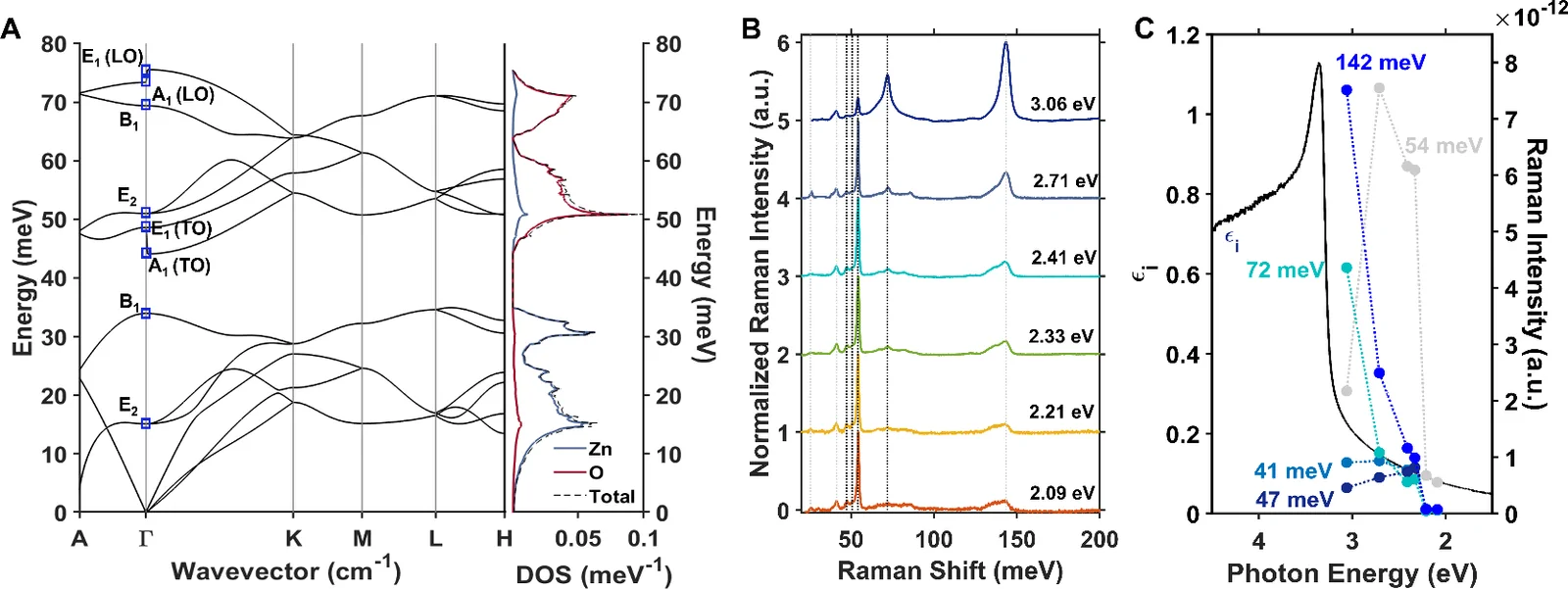
**A)** Computed phonon dispersion curve of ZnO and associated projected vibrational densities of state. The blue squares indicate the phonon modes at Γ. **B)** Stokes resonance Raman profile of polycrystalline ZnO on a gold-plated glass substrate collected with excitation energies from 2.09 to 3.06 eV; each spectrum is internally normalized. The vertical lines follow the Raman peak centers: the black lines are fundamental modes, and the gray lines are second-order modes. Table 1 contains the full peak assignments. **C)** Imaginary dielectric spectrum of ZnO (black) overlaid with Raman intensities corrected for scattering cross section.

Upon comparing the computed phonon modes to the experimental Raman spectra shown in Figure 2B, we find that the experimental spectrum exhibits more peaks than the number of calculated modes (Table 1). The second-order scattering of phonons is a well-established phenomenon in ZnO^18,32–34^ and has been thoroughly investigated with polarization-dependent variable-temperature Raman measurements of a single crystal of ZnO by Cuscó, et al.^32^ They assign these second-order modes to various difference modes, overtones, and combination bands. Here, we focus on the first-order modes and the highest energy second-order mode observed at 142 meV. This second-order mode is assigned to a doubling of the E_1_ (LO) and/or A_1_ (LO) modes, which are nearly identical in energy and could not be resolved from each other in our measurements. These two modes both contribute to the first-order feature observed at 72.7 meV.

**Table 1:** Optical Phonon Modes in Wurtzite ZnO

*Optical Phonon Mode* ^a^	*Raman Peak Center (meV)*	*DFT+U Phonon Energy (meV)*
*E*_2, *low*_	Below detection range	15.13847
*B*_1, *low*_	Raman silent	33.97167
*Second Order*	25.35	-
*Second Order*	41.24	-
*A*_1_ *(transverse)*	47.57	44.10614
*E*_1_ *(transverse)*	50.97	48.6204
*E*_2, *high*_	54.38	51.0195
*B*_1, *high*_	Raman silent	69.39272
*Second Order*	66.55	-
*A*_1_ *(longitudinal)*	72.74	73.36150
*E*_1_ *(longitudinal)*	72.74	75.54109
*Second Order*	86.13	-
*Second Order*	137.44	-
2*A*_1_ *LO,* 2 *E*_1_ *LO*	142.56	-

a Assignments based on refs 30 and 32, which contains a more detailed discussion of the specific assignments of second-order modes.

By varying the excitation photon energy across the imaginary dielectric spectrum of ZnO, we can assess which phonon modes exhibit significant resonance enhancement at the onset of excitonic absorption. Figure 2B plots internally normalized resonance Raman spectra collected with various excitation photon energies. These spectra show that the 54-meV mode (E_2_) is the most intense mode at all excitation photon energies below 3.06 eV; at 3.06 eV the 72.7 meV and 142 meV modes dominate the spectrum. Figure 2C plots the Raman intensity corrected for scattering cross section versus excitation photon energy. It is clear from this plot that, as the excitation photon energy approaches the onset of the excitonic absorption feature, the 72.7-meV and 142-meV modes exhibit the strongest resonance enhancement. Calleja, et al. report Raman spectra of ZnO collected with different excitation sources (*hν* = 2.71 eV (457.9 nm), 2.54 eV (488.0 nm), and 1.91 eV (647.1 nm)), and they also observe that the second-order feature at 142 meV exhibits the strongest resonance enhancement at the highest excitation photon energy.^18^ They suggest that this resonance enhancement may be due to a long-range Frölich interaction.^18^ Similar increases in the intensity of second-order scattering of LO phonons as the photon excitation energy approaches the absorption onset have been observed in ZnTe^35^ and hematite;^8^ in both cases, this pattern of resonance Raman enhancement of specific modes at band-edge absorption energies has been attributed to formation of polaronic states. We note that the linewidths of the second-order features that exhibit resonance enhancement in α-Fe_2_O_3_ and ZnO are broadened compared to the corresponding first-order features. This broadening arises from the participation of phonon modes with a range of momenta in the second-order scattering process (Raman selection rules restrict first-order scattering to Γ-point modes). The observation that resonance enhancement occurs across the broad envelope of the second-order scattering feature implies that the coupling of the A_1_ and E_1_ phonon modes to the exciton in ZnO extends in k-space beyond the Γ-point, which is consistent with polaron formation.

Temperature-dependent optical spectroscopy complements resonance Raman spectroscopy by revealing the extent to which thermally populated phonon modes couple to optical transitions to enable excitation into polaronic states. Figure 3A plots thermal difference optical absorption spectra of ZnO collected above and below room temperature. The vertical dotted line marks the position of the peak center of the exciton absorption in ZnO observed at room temperature. Above room temperature, there is an increase in absorption at the lower energy side of the excitonic absorption, and, conversely, a decrease in absorption is observed below room temperature. Although the temperature-dependent absorbance is shown as a difference relative to room temperature, there are still three peaks that become apparent at low temperature with peak centers at 3.326 eV (a), 3.382 eV (b), and 3.443 eV (c) (Figure 3A). The two higher-energy peak center values match the positions of peaks observed in low-temperature reflection spectra originally reported by Thomas (and later papers).^13,14,16^ The lower energy peak (which decreases in intensity as temperature decreases) reported here is ∼0.05 eV lower than that reported by Thomas, which may be attributed to the fact that his spectrum was measured at 4.2 K, whereas the lowest temperature spectrum collected in this study was at 82 K. The overall similarity of the thermal difference spectra of ZnO reported here for polycrystalline thin films to temperature-dependent reflectivity spectra reported for single-crystalline ZnO demonstrates that the temperature-dependent excitonic nature of this material extends to the polycrystalline thin film form.

**Figure 3:**
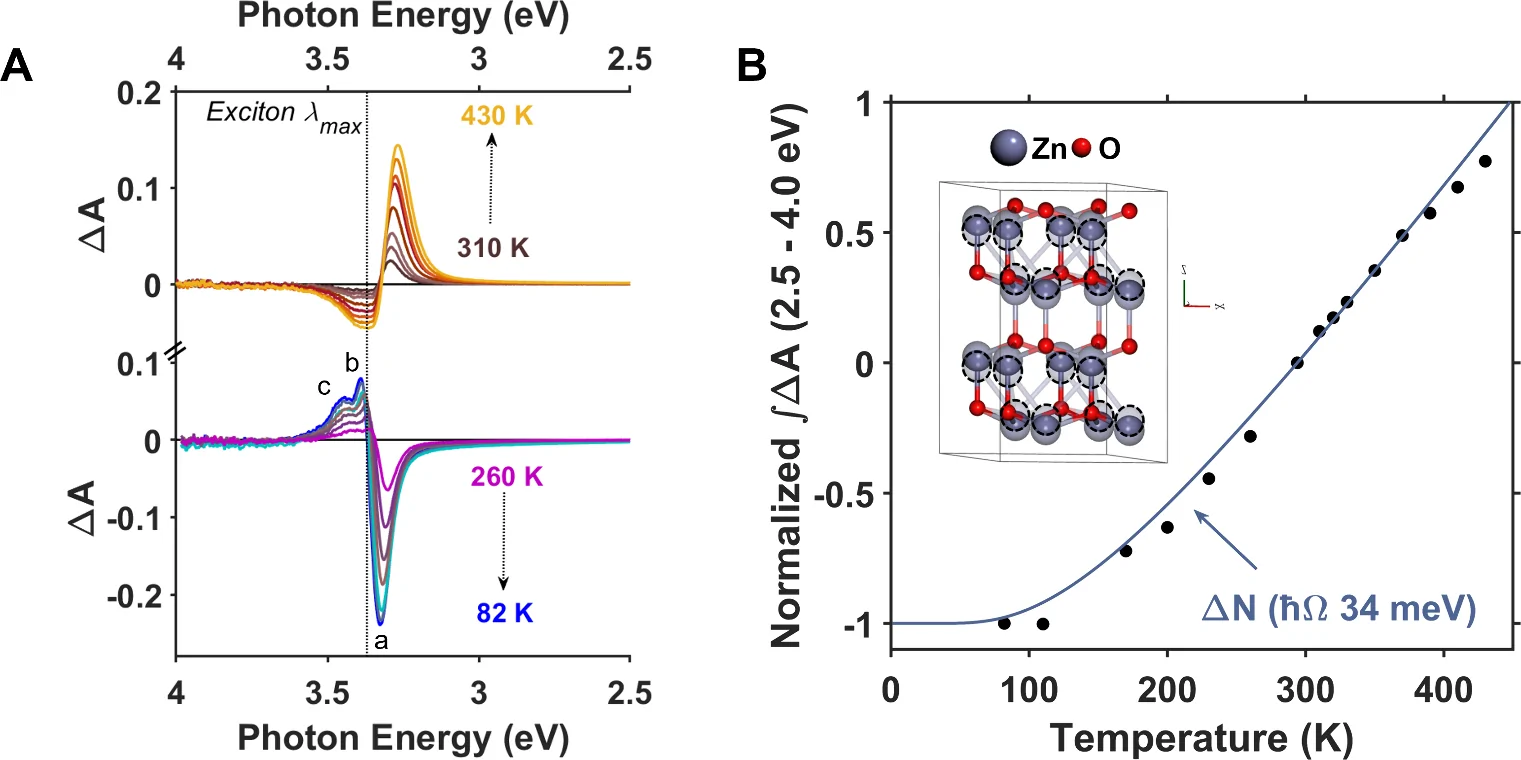
**A)** Thermal difference optical absorption spectra of ZnO collected above room temperature (310–430 K) (top) and below room temperature (82–260 K) (bottom). The vertical dotted line indicates the position of the excitonic peak observed at 3.36 eV in the room-temperature spectrum. **B)** Normalized integrated intensities of thermal difference absorption spectra shown in part (**A**) plotted against the temperatures at which the spectra were collected. The blue line represents a Bose–Einstein model calculated with a phonon energy of 34 meV. The inset shows the atomic displacements corresponding to this phonon mode.

To quantitatively analyze the thermal difference spectra, the integrated intensity of each spectrum was normalized to the integrated intensity obtained at 82 K and plotted versus temperature. Upon comparing this plot to plots of Bose–Einstein functions corresponding to the energies of various first-order phonon modes in ZnO, we find that the Bose–Einstein function corresponding to a phonon energy of 34 meV best matches the experimental data (see Figure S3 in the Supporting Information). The 34-meV phonon mode is not Raman or IR active and involves primarily motion of Zn atoms along the *c*-axis (Figure 3B, inset). The vibrational density of states shown in Figure 2A indicates that this mode sits at the lower edge of the phonon band gap that divides the modes dominated by Zn motion from modes dominated by O motion. This result suggests that thermal activation of excitonic absorption is gated by the activation of oxygen motion. We have identified similar threshold modes from analysis of thermal difference spectra collected for other transition metal oxides that form hole or electron polarons upon photoexcitation, namely, α-Fe_2_O_3_ (hematite), α-Rh_2_O_3_, and Co_3_O_4_.^8–10,12^ These modes do not always coincide with the modes that exhibit the strongest resonance enhancement observed in Raman spectroscopy. Understanding the details of the physical mechanisms that underpin the specific roles of these threshold modes in photoinduced polaron formation in these materials merits significant theoretical effort that is beyond the scope of this study.

To complement the temperature-dependent absorption of ZnO, excitonic emission spectra were also measured as a function of temperature to further assess exciton–phonon coupling. Figure 4A plots the excitonic emission of ZnO collected from 82 K to room temperature using an excitation source with photon energy of 3.49 eV. With decreasing temperature, the position of maximum emission intensity shifts to higher energy, and one broad feature evolves into multiple narrower features. The overall emission intensity also increases with decreasing temperature (Figure 4A). The lower energy features (E ≤ 3.31 eV) that become apparent with decreasing temperature have energetic spacing matching the A_1_-LO/E_1_-LO phonon modes (72 meV), indicative of a Franck–Condon progression. This Franck–Condon behavior has been observed previously,^19,36,37^ and Hsu, et al. similarly find the 72-meV phonon mode modulates the progression.^19^ Furthermore, the Raman spectra shown in Figure 2 demonstrate that the 72.7-meV phonon mode exhibits significant resonance enhancement at the onset of the excitonic absorption feature, corroborating the intrinsic coupling of the A_1_-LO and E_1_-LO phonon modes to excitonic transitions. The highest-energy feature of the temperature-dependent excitonic emission progression at 3.31 eV (b) has previously been assigned to various transitions including a free-to-bound exciton^37^ and a two-electron transition associated with donor-bound excitons.^15,38^ The feature at 3.36 eV (a) is associated with a neutral donor-bound exciton,^15,16^ while the high-energy shoulder around 3.38 eV has been previously assigned to a free exciton transition and in previous reports becomes more spectrally resolved at temperatures below 82 K.^15,16,36–38^

**Figure 4:**
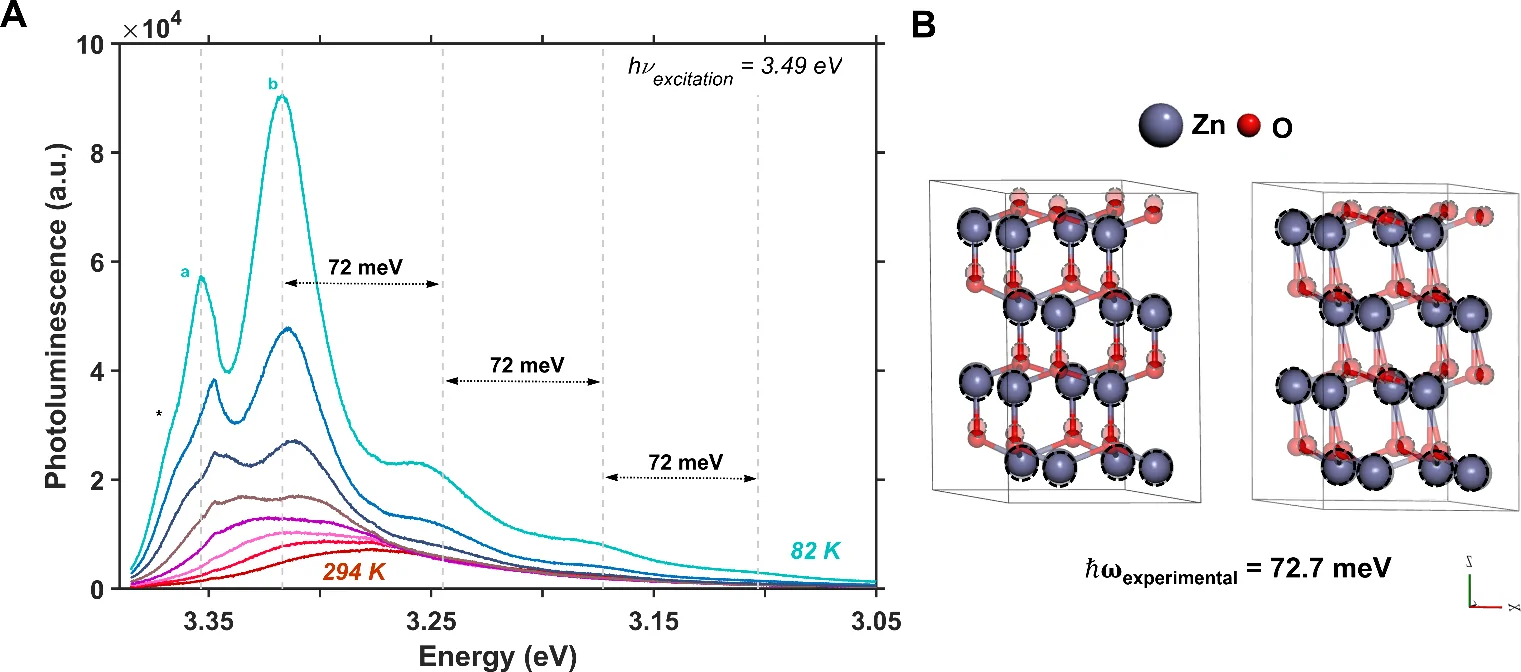
**A)** Variable-temperature excitonic emission of ZnO collected from 82 K to room temperature using an excitation source of 3.49 eV. The differences from peak to peak are labeled on the 82 K spectrum. **B)** Phonon mode displacement vector images associated with the Raman peak observed at 72.7 meV.

The resonance Raman, thermal difference, and temperature-dependent emission data presented here all point to a consistent picture of exciton polaron formation in ZnO mediated by motion within the oxygen sublattice. Both resonance Raman and temperature-dependent emission indicate the strong coupling of the A_1_ (LO) and/or E_1_ (LO) modes to the onset of the optical excitonic transition. These modes correspond to motion of the oxygen atoms parallel or perpendicular (respectively) to the *c*-axis (Figure 4B). Thermal difference absorption spectra suggest that the threshold for thermal activation of excitonic absorption coincides with the energy at which oxygen motion starts to dominate the phonon modes. Taken together, these data are consistent with direct excitation into an exciton polaron state mediated by coupling to thermally populated phonon modes dominated by oxygen motion. This work links the vast body of literature on ZnO photophysics,^13–16,18,19,30–33,39,40^ with which all of the data presented here are consistent, with our recent work on photoinduced small polaron formation in transition metal oxides^4,8–12^ to provide an updated and unified interpretation of how phonon coupling to excitonic transitions mediates formation of an exciton polaron upon photoexcitation of ZnO.

## Experimental and Computational Methods

### Sample Preparation

ZnO thin films were prepared using a sol–gel precursor solution containing 3 mmol of Zn­(II) nitrate tetrahydrate, 4.5 mmol of citric acid monohydrate, and 4.5 mmol of ethylene glycol in 6 mL of ethanol. Aliquots (50 μL) of precursor solution were spin-coated at 5000 rpm onto 1 mm-thick substrates (sapphire or fused quartz) and annealed at 800 °C for 5 min. Multiple layers were deposited in this manner before a final annealing at 800 °C for 24 h followed by cooling to room temperature over 2 h. One annealed aliquot contributes ∼30 nm to film thickness. Crystalline powder samples were prepared from the same precursor solution by first heating 2 mL of the solution in a crucible at 200 °C on a hot plate for 1 h, followed by annealing in a furnace at 800 °C for 24 h. The initial heating step is to minimize the rapid boiling, which can lead to loss of sample in the furnace. Ethylene glycol (1 mL) was added to the powder sample after cooling and the resulting slurry was drop cast onto gold-plated glass. The drop-cast film was then heated at 100 °C until there was a ∼0.5 cm^2^ spot of white coverage adhered to the substrate when held vertically. The powder sample was used for Raman spectra collection.

### Structural Characterization

X-ray diffraction of polycrystalline powders was collected using a Rigaku XtaLAB Dualflex Synergy-S diffraction system with Cu Kα radiation. X-ray diffraction patterns of thin films were collected on a Rigaku 3 kW (Cu Kα radiation) SmartLab system with a chi-phi cradle, cross beam optics, and a HyPix-3000 detector. Scans were performed in 2θ in parallel beam mode with grazing incident angles between 0.5 and 1.5 degrees. Optics included an incident 0.5-degree Soller slit, an incident slit width of 0.5 mm, a length-limiting slit width of 10 mm, and either an initial receiving slit of 1.0 mm and a second receiving slit of 1.1 mm or both receiving slits open, and a parallel slit analyzer of 0.5 mm. The detector was operated in 0D mode. Scans were performed from 5 to 70 degrees in 2θ in steps of 0.01 degree at speeds ranging from 4 to 12 degrees per minute.

### Optical Spectroscopy

Transmission and reflection spectra were collected with an Agilent Cary 7000 spectrometer equipped with a Universal Measurement Accessory from 2460 to 200 nm. The samples were set to a 10° angle, and transmission spectra were collected with the detector at 180°, while reflection spectra were collected with a 20° detector angle. Using a previously reported Fresnel analysis method,^8,41^ the imaginary dielectric spectra of the samples were calculated from transmission and reflection spectra collected at room temperature. Temperature-dependent spectra above room temperature were collected using a home-built setup in which the sample is sandwiched between two donut-shaped ceramic heaters, which allow optical access through their centers. The temperature was controlled using an Omega temperature controller. For spectra collected below room temperature, an APD Cryogenics Inc. model LT-3-110 Heli-Tran cryostat system run at 10^–5^ Torr vacuum with a turbo molecular vacuum pump was used. The cryostat sleeve is equipped with a quartz window for transmission collection and a large sapphire window to accommodate small-angle reflection collection. The transmission spectra collected at variable temperatures were converted to absorbance in postprocessing.

### Raman Spectroscopy

Resonance Raman spectra profiles were collected using CW diode lasers with wavelengths of 594 nm (2.09 eV), 561 nm (2.21 eV), 532 nm (2.33 eV), 515 nm (2.41 eV), 457 nm (2.71 eV), and 405 nm (3.06 eV) and a Princeton Instruments monochromator equipped with a CCD detector. Laser power was kept at <30 mW to limit the possibility of sample heating. The Raman shift axis was corrected with cyclohexane spectra. For Figure 2C, the Raman intensities of the ZnO spectra collected at each evaluated wavelength were corrected for grating efficiency and scattering cross-section at each wavelength using previously reported procedures.^10,11^

### Emission Spectroscopy

The emission spectra of ZnO samples were collected using a CW diode laser with a photon energy of 3.49 eV (355 nm). The emission signal was directed through a 355 nm notch filter and collected with a 1200 gr/mm grating inside a Princeton Instruments monochromator equipped with a CCD detector. The setup is optimized for backscattered Raman collection; therefore, cyclohexane spectra were collected to correct the *x*-axis. For low-temperature measurements (82–260 K), an APD Cryogenics Inc. model LT-3-110 Heli-Tran cryostat system run at 10^–5^ Torr vacuum with a turbo molecular vacuum pump was used.

### Density Functional Theory

The electronic structure of ZnO was computed using density functional theory (DFT), performed using the pseudopotential plane wave package implemented in Quantum ESPRESSO.^42–44^ For the purpose of this study, previously reported Hubbard *U* parameters (12.0 eV for Zn and 6.5 eV for O)^31,45^ were applied to obtain phonon displacement vectors at Γ, phonon bands, and phonon densities of state. Although using hybrid functionals has been shown to be critical for accurately calculating electron–phonon coupling,^28^ we find that the energies of first-order Raman modes computed with DFT+*U* agree well with experiment. Additionally, our reported phonon bands and densities of state match those calculated with hybrid-functional approaches.^46^ For the DFT+*U* calculations of ZnO, a high plane wave cutoff energy of 1000 eV was used to ensure the total energy and total interatomic forces were converged to 10 meV and 0.10 meV/Å, respectively. ZnO was sampled over a 6 × 6 × 6 k-point grid. Both the Perdew, Burke, and Ernzerh exchange–correlation functional revised for solids (PBEsol)^47,48^ and Optimized Norm-Conserving Vanderbilt (ONCV)^49,50^ method for pseudopotentials were implemented throughout this work. Iterative geometric relaxations were performed using the Broyden–Fletcher–Goldfarb–Shanno (BFGS) algorithm.^51–53^ Phonon modes of ZnO were calculated with density functional perturbation theory (DFPT) using the PHonon code implemented in Quantum ESPRESSO.^42–44^ Starting with the relaxed ground-state configuration, dynamical matrices were evaluated at the high-symmetry point Γ. The vibrational density of states was determined from evaluating a uniform 3 × 3 × 3 grid of q-points followed by finer sampling via Fourier interpolation on a denser *q*-grid and then a defined path for the vibrational band structure.

## Supplementary Material



## Notes

Transmission and reflection spectra used in Fresnel analysis, displacement vectors for each first-order phonon mode, and comparison of the temperature-dependent thermal difference intensities to various Bose–Einstein functions (PDF)

## References

[1] Lany S. (2015). Semiconducting
Transition Metal Oxides. J. Phys.: Condens.
Matter.

[2] Franchini C., Reticcioli M., Setvin M., Diebold U. (2021). Polarons in Materials. Nat. Rev. Mater..

[3] Cheng C., Zhou Z., Long R. (2023). Time-Domain
View of Polaron Dynamics
in Metal Oxide Photocatalysts. J. Phys. Chem.
Lett..

[4] Craddock E. P., Knowles K. E. (2026). Signatures of Photogenerated Small
Polarons in Transition
Metal Oxides. Chem. Commun..

[5] Landau L. D. (1933). Electron
Motion in Crystal Lattices. Phys. Z. Sowjetunion.

[6] Pekar S. I. (1946). Local Quantum
States of Electrons in an Ideal Ion Crystal. Zh. Eksp. Teor. Fiz..

[7] Landau L. D., Pekar S. I. (1948). Effective Mass of Polaron. Zh.
Eksp. Teor. Fiz..

[8] Shelton J. L., Knowles K. E. (2021). Thermally Activated Optical Absorption into Polaronic
States in Hematite. J. Phys. Chem. Lett..

[9] Shelton J. L., Knowles K. E. (2022). Polaronic Optical Transitions in Hematite (α-Fe_2_O_3_) Revealed by First-Principles Electron–Phonon
Coupling. J. Chem. Phys..

[10] Craddock E. P., Shelton J. L., Ruggiero M. T., Knowles K. E. (2025). Local Coordination
Geometry within Cobalt Spinel Oxides Mediates Photoinduced Polaron
Formation. Chem. Sci..

[11] Craddock E. P., Brennessel W. W., Ruggiero M. T., Knowles K. E. (2026). Correlating Composition
and Cation Inversion in Ternary Iron Spinel Oxides with Localization
of Photoexcited States. J. Am. Chem. Soc..

[12] Craddock E. P., Knowles K. E. (2026). Assessing the Influence of *d* -Orbital
Radius on Formation of Localized Photogenerated States in Corundum
Metal Oxides. J. Chem. Phys..

[13] Thomas D. G. (1960). The Exciton
Spectrum of Zinc Oxide. J. Phys. Chem. Solids.

[14] Park Y. S., Litton C. W., Collins T. C., Reynolds D. C. (1966). Exciton
Spectrum
of ZnO. Phys. Rev..

[15] Reynolds D. C., Look D. C., Jogai B., Litton C. W., Collins T. C., Harsch W., Cantwell G. (1998). Neutral-Donor–Bound-Exciton
Complexes in ZnO Crystals. Phys. Rev. B.

[16] Reynolds D. C., Look D. C., Jogai B., Litton C. W., Cantwell G., Harsch W. C. (1999). Valence-Band Ordering in ZnO. Phys. Rev. B.

[17] Scott J. F. (1970). UV Resonant
Raman Scattering in ZnO. Phys. Rev. B.

[18] Calleja J. M., Cardona M. (1977). Resonant Raman Scattering in ZnO. Phys. Rev. B.

[19] Hsu H.-C., Hsieh W.-F. (2004). Excitonic Polaron and Phonon Assisted
Photoluminescence
of ZnO Nanowires. Solid State Commun..

[20] Lany S., Zunger A. (2009). Polaronic Hole Localization and Multiple Hole Binding
of Acceptors in Oxide Wide-Gap Semiconductors. Phys. Rev. B.

[21] Antonius G., Chan Y.-H., Louie S. G. (2020). Polaron Spectral Properties in Doped
ZnO and SrTiO_3_ from First Principles. Phys. Rev. Res..

[22] Hua F., Zhang Z., Wang Z., Liu Y., Gong C., Hu C., Zhou Y., Liang W. (2026). Polaronic-Distortion-Driven
Enhancement
of Excitonic Auger Recombination in ZnO Nanoparticles. J. Phys. Chem. Lett..

[23] Penfold T. J., Szlachetko J., Santomauro F. G., Britz A., Gawelda W., Doumy G., March A. M., Southworth S. H., Rittmann J., Abela R., Chergui M., Milne C. J. (2018). Revealing
Hole Trapping in Zinc Oxide Nanoparticles by Time-Resolved X-Ray Spectroscopy. Nat. Commun..

[24] Foglia L., Vempati S., Tanda Bonkano B., Gierster L., Wolf M., Sadofev S., Stähler J. (2019). Revealing
the Competing Contributions
of Charge Carriers, Excitons, and Defects to the Non-Equilibrium Optical
Properties of ZnO. Struct. Dyn..

[25] Liu Q., Chen H., Wang Z., Weng Y. (2021). Observation of the
Polaron Excited State in a Single-Crystal ZnO. J. Phys. Chem. C.

[26] Deinert J.-C., Wegkamp D., Meyer M., Richter C., Wolf M., Stähler J. (2014). Ultrafast
Exciton Formation at the ZnO (101̅0)
Surface. Phys. Rev. Lett..

[27] Hendry E., Koeberg M., Bonn M. (2007). Exciton and
Electron-Hole Plasma
Formation Dynamics in ZnO. Phys. Rev. B.

[28] Sezen H., Shang H., Bebensee F., Yang C., Buchholz M., Nefedov A., Heissler S., Carbogno C., Scheffler M., Rinke P., Wöll C. (2015). Evidence for
Photogenerated Intermediate
Hole Polarons in ZnO. Nat. Commun..

[29] Pollak, F. H. Exciton-Optical Phonon Interaction in Reduced Dimensional Systems: Temperature Dependence of the Linewidth. In Phonons in Semiconductor Nanostructures; Leburton, J.-P. , Pascual, J. , Torres, C. S. , Eds.; Springer Netherlands: Dordrecht, 1993; pp 341–351 10.1007/978-94-011-1683-1_33.

[30] Arguello C. A., Rousseau D. L., Porto S. P. S. (1969). First-Order
Raman Effect in Wurtzite-Type
Crystals. Phys. Rev..

[31] Calzolari A., Nardelli M. B. (2013). Dielectric Properties
and Raman Spectra of ZnO from
a First Principles Finite-Differences/Finite-Fields Approach. Sci. Rep..

[32] Cuscó R., Alarcón-Lladó E., Ibáñez J., Artús L., Jiménez J., Wang B., Callahan M. J. (2007). Temperature
Dependence of Raman Scattering in ZnO. Phys.
Rev. B.

[33] Damen T. C., Porto S. P. S., Tell B. (1966). Raman Effect in Zinc Oxide. Phys. Rev..

[34] Rajalakshmi M., Arora A. K., Bendre B. S., Mahamuni S. (2000). Optical Phonon Confinement
in Zinc Oxide Nanoparticles. J. Appl. Phys..

[35] Schmidt R.
L., McCombe B. D., Cardona M. (1975). Resonant First- and Second-Order
Raman Scattering in ZnTe. Phys. Rev. B.

[36] Misra P., Sharma T. K., Kukreja L. M. (2009). Temperature Dependent Photoluminescence
Processes in ZnO Thin Films Grown on Sapphire by Pulsed Laser Deposition. Curr. Appl. Phys..

[37] Zhao Q. X., Willander M., Morjan R. E., Hu Q.-H., Campbell E. E. B. (2003). Optical
Recombination of ZnO Nanowires Grown on Sapphire and Si Substrates. Appl. Phys. Lett..

[38] Greene L. E., Yuhas B. D., Law M., Zitoun D., Yang P. (2006). Solution-Grown
Zinc Oxide Nanowires. Inorg. Chem..

[39] Birman J.
L. (1963). Theory
of Infrared and Raman Processes in Crystals: Selection Rules in Diamond
and Zincblende. Phys. Rev..

[40] Zhang R., Yin P.-G., Wang N., Guo L. (2009). Photoluminescence and
Raman Scattering of ZnO Nanorods. Solid State
Sci..

[41] Barybin A., Shapovalov V. (2010). Substrate
Effect on the Optical Reflectance and Transmittance
of Thin-Film Structures. Int. J. Opt..

[42] Giannozzi P., Baroni S., Bonini N., Calandra M., Car R., Cavazzoni C., Ceresoli D., Chiarotti G. L., Cococcioni M., Dabo I., Dal Corso A., De Gironcoli S., Fabris S., Fratesi G., Gebauer R., Gerstmann U., Gougoussis C., Kokalj A., Lazzeri M., Martin-Samos L., Marzari N., Mauri F., Mazzarello R., Paolini S., Pasquarello A., Paulatto L., Sbraccia C., Scandolo S., Sclauzero G., Seitsonen A. P., Smogunov A., Umari P., Wentzcovitch R. M. (2009). QUANTUM
ESPRESSO: A Modular and Open-Source Software Project for Quantum Simulations
of Materials. J. Phys.: Condens. Matter.

[43] Giannozzi P., Andreussi O., Brumme T., Bunau O., Buongiorno
Nardelli M., Calandra M., Car R., Cavazzoni C., Ceresoli D., Cococcioni M., Colonna N., Carnimeo I., Dal Corso A., De Gironcoli S., Delugas P., DiStasio R. A., Ferretti A., Floris A., Fratesi G., Fugallo G., Gebauer R., Gerstmann U., Giustino F., Gorni T., Jia J., Kawamura M., Ko H.-Y., Kokalj A., Küçükbenli E., Lazzeri M., Marsili M., Marzari N., Mauri F., Nguyen N. L., Nguyen H.-V., Otero-de-la-Roza A., Paulatto L., Poncé S., Rocca D., Sabatini R., Santra B., Schlipf M., Seitsonen A. P., Smogunov A., Timrov I., Thonhauser T., Umari P., Vast N., Wu X., Baroni S. (2017). Advanced Capabilities
for Materials Modelling with Quantum ESPRESSO. J. Phys.: Condens. Matter.

[44] Giannozzi P., Baseggio O., Bonfà P., Brunato D., Car R., Carnimeo I., Cavazzoni C., De Gironcoli S., Delugas P., Ferrari Ruffino F., Ferretti A., Marzari N., Timrov I., Urru A., Baroni S. (2020). Q uantum ESPRESSO
toward the Exascale. J. Chem. Phys..

[45] Calzolari A., Ruini A., Catellani A. (2011). Anchor Group
versus Conjugation:
Toward the Gap-State Engineering of Functionalized ZnO(101̅0)
Surface for Optoelectronic Applications. J.
Am. Chem. Soc..

[46] Wróbel J., Kurzydłowski K. J., Hummer K., Kresse G., Piechota J. (2009). Calculations of ZnO
Properties Using the Heyd-Scuseria-Ernzerhof
Screened Hybrid Density Functional. Phys. Rev.
B.

[47] Perdew J. P., Burke K., Ernzerhof M. (1996). Generalized Gradient Approximation
Made Simple. Phys. Rev. Lett..

[48] Perdew J. P., Ruzsinszky A., Csonka G. I., Vydrov O. A., Scuseria G. E., Constantin L. A., Zhou X., Burke K. (2008). Restoring the Density-Gradient
Expansion for Exchange in Solids and Surfaces. Phys. Rev. Lett..

[49] Hamann D. R. (2013). Optimized
Norm-Conserving Vanderbilt Pseudopotentials. Phys. Rev. B.

[50] Van
Setten M. J., Giantomassi M., Bousquet E., Verstraete M. J., Hamann D. R., Gonze X., Rignanese G.-M. (2018). The PseudoDojo:
Training and Grading a 85 Element Optimized Norm-Conserving Pseudopotential
Table. Comput. Phys. Commun..

[51] Fletcher, R. Practical Methods of Optimization, 2nd ed.; Wiley: Chichester, New York, 1987.

[52] Billeter S. R., Turner A. J., Thiel W. (2000). Linear Scaling Geometry Optimisation
and Transition State Search in Hybrid Delocalised Internal Coordinates. Phys. Chem. Chem. Phys..

[53] Billeter S. R., Curioni A., Andreoni W. (2003). Efficient
Linear Scaling Geometry
Optimization and Transition-State Search for Direct Wavefunction Optimization
Schemes in Density Functional Theory Using a Plane-Wave Basis. Comput. Mater. Sci..

