# Supplementary material for: Evidence of Direct Exciton Polaron Formation upon Photoexcitation in Wurtzite ZnO
Source: J Phys Chem Lett. 2026 Sep 25;17(40):11374–80. doi: 10.1021/acs.jpclett.6c02424 (PMC13656779; doi:10.1021/acs.jpclett.6c02424)
Supplement: Supplementary file 1 [file jz6c02424_si_001.pdf]

Supporting Information

Evidence of Direct Exciton Polaron Formation Upon Photoexcitation in Wurtzite ZnO

Erica P. Craddock and Kathryn E. Knowles*

Department of Chemistry, University of Rochester, Rochester, NY 14627, USA

*Corresponding Author E-mail: kknowles@ur.rochester.edu

The Fresnel analysis used to calculate the imaginary dielectric spectrum shown in Figure 1 required the transmission and reflection of three films of varying thickness and substrate,[1,2] which are shown in Figure S1A. The corresponding powder X-ray diffraction patterns are shown in Figure S1B.

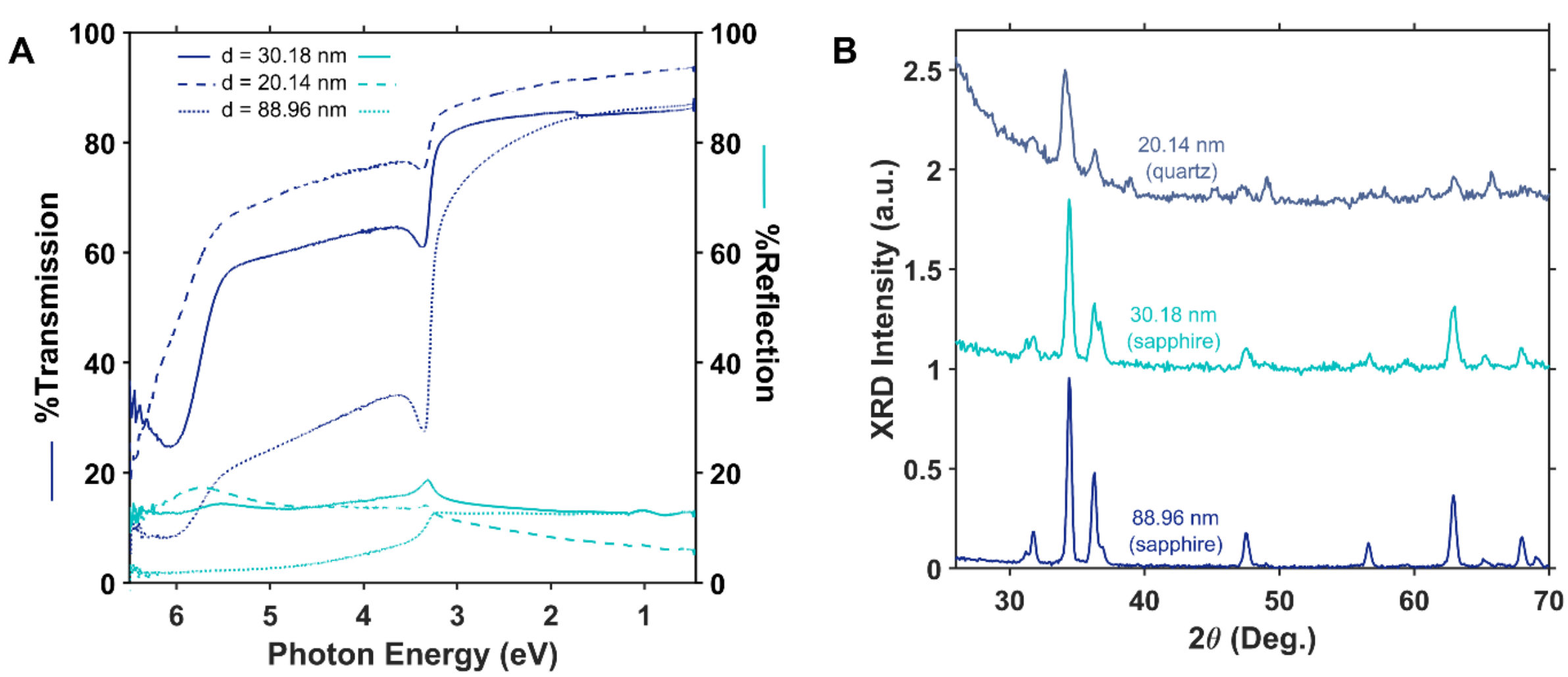

Figure S1. A) Transmission and reflection spectra of all ZnO thin films used for Fresnel analysis and **B)** corresponding powder X-ray diffraction patterns of the same samples.

The optical phonon displacement vectors computed with DFT+U are shown in Figure S2 aligned energetically with the vibrational band structure and density of states.

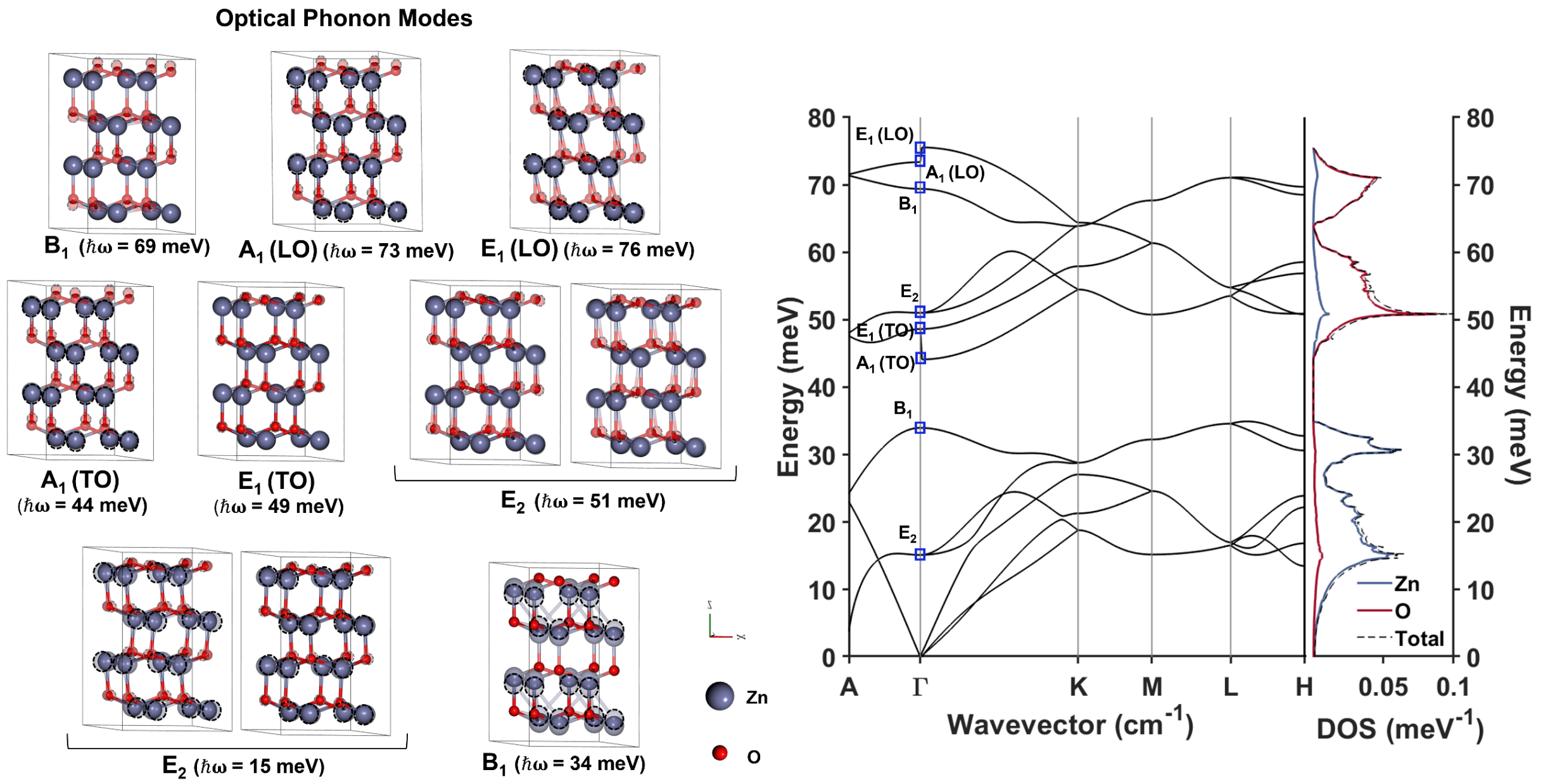

Figure S2. Phonon dispersion curve of ZnO and associated vibrational densities of state with all optical phonon mode displacement vectors aligned vertically from lowest (bottom) to highest (top) energy. The blue squares indicate the phonon modes at Γ represented as vector displacement images on the left.

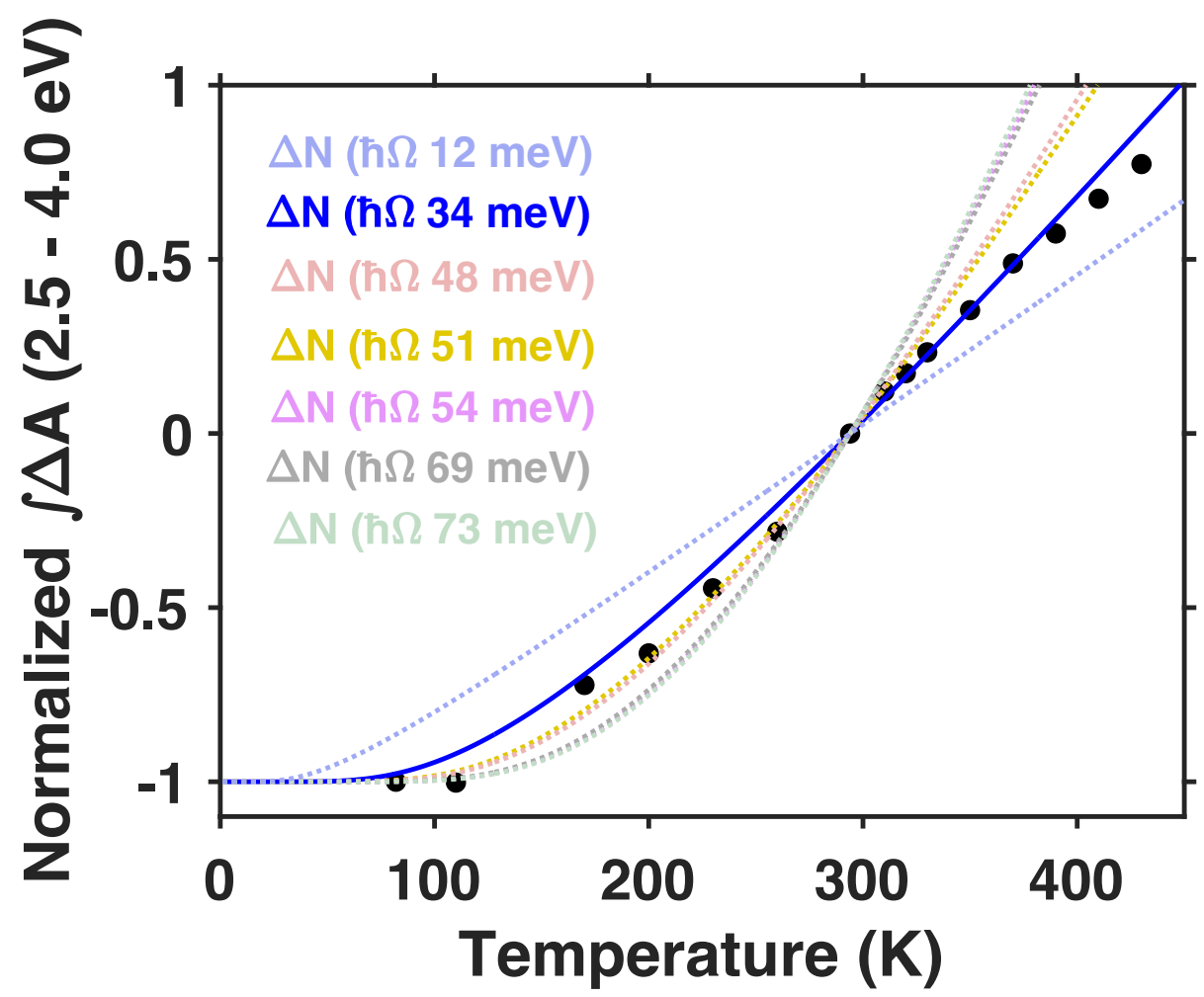

Figure S3. Plot of the integrated intensities of thermal difference spectra versus temperature overlaid with Bose-Einstein functions corresponding to the energies of each of the fundamental phonon modes of wurtzite ZnO. The solid blue line represents the Bose-Einstein function for a phonon energy of 34 meV, which is shown in Figure 3 of the main text.

References

(1) Barybin, A.; Shapovalov, V. Substrate Effect on the Optical Reflectance and Transmittance of Thin-Film Structures. *Int. J. Opt.* **2010**, *2010* (1), 137572.

(2) Shelton, J. L.; Knowles, K. E. Thermally Activated Optical Absorption into Polaronic States in Hematite. *J. Phys. Chem. Lett.* **2021**, *12* (13), 3343–3351.
